# How to better consider and understand interindividual variability in response to polyphenols in clinical trials

**DOI:** 10.3389/fnut.2024.1522516

**Published:** 2024-12-18

**Authors:** Christine Morand

**Affiliations:** Université Clermont Auvergne, INRAE, UNH, Clermont-Ferrand, France

**Keywords:** polyphenols, interindividual variability, clinical trials, ADME, (cardio)metabolic response, nutrigenomics, gut microbiota, precision nutrition

## Introduction

Polyphenols are the largest family of bioactive compounds in plant foods and represent a significant component of our diet. Evidence from large cohort studies and experimental research consistently indicates beneficial effects of polyphenol intake on cardiometabolic health (1–3). However, randomized controlled trials (RCTs) investigating polyphenol effects on cardiometabolic outcomes often yield mixed or inconclusive results, which can undermine the perceived efficacy of polyphenols in health interventions (4). A primary cause of these inconsistencies appears to be significant interindividual variability in response to polyphenols, distinguishing “responders” from “non-responders” within the same study cohort (5). For instance, a one-year flavonoid intervention study in postmenopausal women demonstrated notable variability in urinary flavonoid excretion rates, categorizing participants as “poor” or “high” excretors (6). This interindividual variability in flavonoid metabolism correlated with differences in insulin response, highlighting that variations in metabolic capacity can significantly impact physiological outcomes. However, other RCTs with different polyphenols revealed that vascular responses (e.g., blood pressure, endothelial function) varied across study populations depending on age and sex, even when bioavailability measures were similar (7–9). This heterogeneity in clinical trials poses challenges for meta-analyses, as seen when wide standard deviations and high statistical heterogeneity obscure the effects in specific populations and hamper to draw consistent conclusions about polyphenols effects (10–12). Interindividual variability in response may stem from differences in ADME processes (absorption, distribution, metabolism, excretion) or from varied responsiveness of cellular and molecular targets critical to the cardiometabolic effects of polyphenols. Key determinants likely include genetic background, age, sex, health status, and gut microbiota composition, but yet their individual contributions and interactions are poorly understood.

## Efforts to understand interindividual variability in polyphenol response

The European scientific network COST POSITIVe undertook a systematic analysis to map interindividual variability in polyphenol responses and get insights into why polyphenols are effective in some individuals and less so in others (13). This analysis revealed a significant variability across polyphenol ADME capacities. For most polyphenol classes, this variation appears primarily driven by gut microbiota composition and functionality which play a central role in converting food-derived phenolics into bioactive metabolites (14, 15). POSITIVe highlighted the need for further exploration of other determinants, such as age, sex, and genetic background, where information remains limited. POSITIVe also underscored the value of metabotyping individuals for better predictive power, advocating for standardized approaches, such as using polyphenol supplements for challenge tests and advancing mass spectrometry-based metabolomic profiling (14). The POSITIVe network also conducted meta-analyses of published RCTs to examine variability in cardiometabolic responses to different polyphenol classes, employing subgroup and meta-regression analyses to identify factors (e.g., age, sex, BMI, health status) that may affect the biological response to polyphenol consumption. Although overweight individuals or those with cardiovascular risk factors appeared to respond more consistently, findings were inconsistent across polyphenol types and health outcomes, highlighting the need for further investigation (16).

## Strategies for addressing interindividual variability in clinical trials

The current data on potential determinants of interindividual variability in response to polyphenols largely stems from *post-hoc* analyses, as few trials are designed specifically to capture this variability. Addressing interindividual variability in polyphenol research requires to use a flexible and tailored approach. Broadly, the suitable strategies can be divided into: (1) data-driven methods, which focus on the collection and analysis of rich datasets to characterize individual variability, and (2) enhanced experimental designs, which aim to control for or leverage this variability in clinical trials. Depending on the study's objectives, researchers can apply these strategies individually or in combination.The proposed approaches and their potential contributions to advancing the field are detailed below.

### Data-driven methods

*A thorough baseline assessment* of study participants constitutes the foundation of data driven methods by providing comprehensive insights into individual characteristics such as age, sex, genetics, gut microbiota composition, health status and lifestyle factors. These data help contextualize individual responses and identify potential confounding factors. These baseline data allow for detailed correlation analyses between rich datasets and metabolic profiles or health outcomes, offering more personalized interpretations of response variability.

Building on the baseline assessment, *metabotyping* offers a practical way to stratify individuals into meaningful subgroups based on their metabolic capacities toward polyphenols. The metabotypes characterized by a simple dichotomy between “producer” vs. “non-producer” of specific polyphenol derived gut metabolites are quite limited (17). The most common situation is the production of all the phenolic metabolites of a catabolic pathway, but in different proportions depending on the individual (18). Accurately capturing the range of possible metabotypes requires standardized methodological workflows. Comprehensive metabolomic profiling, using techniques like mass spectrometry, enables high-resolution assessment of metabolites in biological fluids. However, the development of advanced standardized methodological and statistical tools is essential for delineating the full spectrum of metabotypes. By categorizing participants into diverse metabotypes, researchers gain a nuanced view of individual metabolic responses and can develop more precise predictions of polyphenol efficacy that are requested to move toward personalized nutrition approaches (19).

While metabotyping provides practical groupings of individuals based on metabolic profiles, the integration of *omics technologies*—such as genomics, epigenomics, transcriptomics, proteomics, metabolomics, and metagenomics—into clinical trials with polyphenols can comprehensively illuminate factors driving interindividual variability and allow to understand how different biological systems interact to produce varying responses to polyphenols. For example, genomics reveals genetic variations impacting polyphenol metabolism, such as polymorphisms in genes encoding conjugative enzymes (e.g., UGT1A1, SULT1A1, COMT) or cell transporters. Epigenomics explores how environmental factors modify responses to polyphenol metabolites. Transcriptomics aids in understanding the influence of polyphenols on cellular pathways, while proteomics identifies specific proteins or enzymes that are over- or under-expressed in individuals. Metabolomics differentiates metabotypes by profiling specific metabolites, while metagenomics characterizes gut bacterial communities responsible for converting polyphenols into bioactive compounds. Machine learning and big data analytics are essential for analyzing these large, complex datasets, identifying response patterns, and creating predictive models of interindividual variability.

In addition to these data-driven methods useful to characterize individual variability some experimental designs can be applied to control and manage this variability effectively in clinical trials.

### Experimental designs

*Stratified randomization* distributes participants based on key variables likely to influence polyphenol metabolism or biological response. To that end, data from baseline assessments or metabotyping can be used to balance key factors across study arms. In particular, this approach could involve stratification by genetic and microbiome profiles, where, participants are grouped based on some genetic polymorphisms and gut microbiota composition. For instance, genetic polymorphisms in phase-2 conjugative enzymes and polyphenol transpoters could influence the profile of circulating metabolites and thereby the bioactivity of polyphenol metabolites, while gut microbiota composition could alter polyphenol metabolism and bioactive metabolite production. Stratifying participants by these characteristics prior to randomization ensures that individuals with distinct metabolic capacities are evenly distributed across study arms. The use of other phenotypic variables such as age, sex, metabolic health, and lifestyle parameters could further refine stratification, enabling more precise exploration of differential responses to polyphenols and clearer identification of responsive subgroups. Stratified randomization minimizes variability and facilitates the identification of specific factors driving interindividual differences.

Used alone or in complement to stratified randomization, *crossover designs* offer an additional way to reduce variability by allowing participants to serve as their own control. Indeed, a crossover design minimizes baseline discrepancies, such as differences in gut microbiota composition, and clarifies intervention-specific effects. This design is particularly effective for acute or short-term studies, as it reduces the influence of between-subject differences, thereby clarifying intervention-specific effects.

While crossover designs address group-level variability, *N-of-1 trials* shift the focus to capturing individual responses in a highly personalized manner. Indeed these trials which not consider average responses are valuable for assessing the effects of a specific intervention on each participant. This approach allows for response measurement over time, reducing intra- and interindividual variability as participants undergo multiple intervention and control periods. If N-of-1 trials support a personalized medicine approach, where each intervention is tailored to the individual's unique response profile, they have been yet poorly considered in the field of polyphenols. However, this method can be particularly useful in polyphenol trials as it captures unique response variations that may be masked in group-based designs. Of note, the unique N-of-1 trial conducted with polyphenols used cocoa flavanols and it revealed wide variability in blood pressure responses, with some individuals responding inconsistently or not at all, and also identified the baseline blood pressure as a major determinant of the vascular response (20). Aggregating N-of-1 data across participants with shared genetic or phenotypic characteristics can reveal response clusters and guide more personalized polyphenol interventions.

Finally, *adaptive trial designs* integrate insights from both data-driven methods and experimental designs, allowing for real-time adjustments to refine study protocols. Of note, adaptive trial designs are increasingly recommended methods to enhance intervention efficacy in clinical nutrition research (21). They allow protocol modifications during the study, based on interim data analyses, without compromising study validity or integrity. This flexibility enhances intervention effectiveness by dynamically adjusting factors -such as participant selection, dosage, outcome measures or study duration- based on real-time data. For instance, adaptations could involve further stratification of participants according to response profiles (responders vs. non-responders) in the study's early stages. This is particularly useful for trials focusing on interindividual variability, as it enables early identification of differing responses and refines interventions to maximize impact on responsive subgroups. Using interim analyses for protocol adjustments ensures that trials can capture the nuances of polyphenol responses and adapt the study design to meet specific research objectives.

Table 1 provides a synthesized overview of the proposed strategies, highlighting their specific contributions to addressing interindividual variability in polyphenol responses. The distinction between data-driven methods and experimental designs highlights the complementary nature of these approaches and how they can be applied flexibly depending on the study objectives and available resources. By combining these approaches as needed, researchers can advance polyphenol research and better address interindividual variability in clinical trials.

**Table 1:** Overview of recommended approaches to address interindividual variability in polyphenol response.

**Category/approach**	**Description**	**Strengths**	**Limitations**	**Applications**
**Data driven methods**				
Baseline assessment	Extensive evaluation of intrinsic and extrinsic individual characteristics	Enables personalized insights via baseline data correlation	Resource intensive, could require advanced analytics	Key for all study designs and stratification strategies
Metabotyping	Stratifies individuals into subgroups based on shared metabolic capacities toward polyphenols	Distinguishes metabolic response patterns; aids subgroup analyses	Requires standardized tools and procedures, complex data analysis	Stratification of populations by metabolic profiles for subgroup-specific interventions
Omics technologies	Uses multi-omics (genomics, metagenomics, etc.) to study factors driving variability	Reveals complex response drivers and enables predictive modeling	High cost; technical expertise required, big data processing and machine learning capabilities	Precision nutrition requiring detailed, multi-layered insights
**Experimental designs**				
Stratified randomization	Groups participants by baseline characteristics before randomization	Balances key factors across study arms; enhances detection of variability drivers	Requires clear stratification criteria	Ideal when predefined factors are known to influence polyphenol response
Crossover design	Allows participants to serve as their own control over sequential intervention periods	Controls baseline differences, reducing variability between subjects	May have carry over effects; duration constraints	Effective for interventions with expected rapid effects (e.g. bioavailability, acute studies)
N-of-1 trials	Focuses on individual-specific responses across multiple intervention and control phases	Captures unique response profiles; minimizes interindividual variability	Resource-intensive with limited generalizability; requires advanced satistical methods	Best for personalized studies capturing unique responses in variable contexts like polyphenols interventions
Adaptive trial design	Allows real-time protocol adjustments based on interim data analyses	Optimizes study relevance by adjusting for early response trends, target responder groups	Requires sophisticated statistical design; ethical considerations	Longitudinal studies with interim analyses for subgroup refinement

## Discussion

Interindividual variability in response to polyphenols represents both a challenge and an opportunity in clinical nutrition research. The observed differences in absorption, metabolism, and physiological effects underscore the necessity of transitioning from a one-size-fits-all approach to a more personalized framework. The complementary nature of data-driven methods and experimental designs is key to addressing this variability. While data-driven methods provide the foundation for characterizing variability through baseline assessments, metabotyping, and omics technologies, enhancing experimental designs operationalize these insights to refine study protocols and outcomes. By incorporating advanced study designs, omics technologies and stratification strategies, researchers can both better understand the biological and molecular underpinnings of polyphenol efficacy and manage variability effectively. The use of these strategies will ultimately facilitate more tailored and effective nutritional interventions. Moreover, metabotyping and omics technologies offer unprecedented opportunities to uncover response patterns and stratify populations based on metabolic and biological profiles. By integrating these methods into clinical trials, researchers can identify responsive subgroups and develop tailored interventions that maximize the efficacy of polyphenols for specific populations. Also, the integration of innovative trial designs, such as adaptive and N-of-1 approaches, highlights the potential for real-time data analysis and response-driven adjustments that enhance trial relevance and impact.

Future research should prioritize collaboration across disciplines and foster data sharing, aiming to develop predictive models of polyphenol responses that accommodate individual variability. Leveraging machine learning and big data analytics will be crucial for integrating complex datasets and translating findings into actionable recommendations for public health. Embracing these innovative methodologies will not only refine our understanding of polyphenols' cardiometabolic benefits but also pave the way for the development of precise nutrition strategies that maximize health outcomes for diverse populations. The integration of these methodologies into clinical trial practices could transform the field of precision nutrition, making polyphenol-based interventions more impactful. Such advances will undoubtedly strengthen the evidence base for polyphenol efficacy in public health.
